# Not Every Headache Warrants a Head CT: A Recurrent Headache Unveiling H. Pylori-Positive MALToma

**DOI:** 10.1155/carm/1773577

**Published:** 2025-02-11

**Authors:** Rebal Nahas, Serena Khoury, Emanuel-Youssef Dib, Karam Karam, Elias Fiani

**Affiliations:** ^1^Department of Internal Medicine, University of Balamand, Beirut, Lebanon; ^2^Department of Gastroenterology, University of Balamand, Beirut, Lebanon

**Keywords:** case report, eradication therapy, headaches, Helicobacter pylori, MALToma

## Abstract

*Helicobacter pylori (H. pylori)* is a urease-producing bacterium that has a tendency to colonize the gastric mucosa. *H. pylori* can cause atrophic gastritis and gastric intestinal metaplasia (GIM). *H. pylori* has also been associated with MALT lymphoma, which is an extranodal marginal zone lymphoma. The gold standard for the diagnosis of *H. pylori* is histopathological analysis from biopsied gastric mucosa. MALT lymhoma can have a wide range of clinical manifestations, such as epigastric pain, iron-deficiency anemia, and overt upper gastrointestinal (GI) bleeding. MALT lymphoma has been rarely associated with headaches. We describe a case of *H. pylori*-positive MALToma manifesting as epigastric pain occurring concomitantly with throbbing headaches; hence, headache can be a heralding symptom for the diagnosis of MALToma.

**Summary**

• The importance of considering GI pathology in patients presenting with unexplained headaches
• Headache can be a heralding symptom for the diagnosis of MALToma
• Treatment of *H. pylori* for infected patients suffering from migraine has favorable outcome in decreasing the severity of the migraines

## 1. Introduction

*H. pylori* is a Gram-negative, urease-producing, helical, and flagellated organism that has a predilection for the gastric mucosa. *H*. *pylori* can cause atrophic gastritis and GIM, which are premalignant conditions. Left untreated, GIM can progress into gastric dysplasia and eventually into gastric adenocarcinoma. Therefore, a prompt diagnosis and timely management of *H. pylori* are warranted for a complete eradication of the organism. The gold standard for the diagnosis of *H. pylori* is histopatholgical analysis from gastric mucosal biopsies. The mainstay treatment of *H. pylori* consists of antibiotics therapy along with proton pump inhibitors (PPIs). *H*. *pylori* can engender the development of MALToma. Symptoms of MALT lymphoma can range from epigastric pain, fevers, night sweats, and weight loss to upper gastrointestinal bleeding. However, we herein present a case of *H*. *pylori*-positive MALToma manifesting as epigastric pain in conjunction with recurrent episodic throbbing headaches. Complete resolution of headaches was attained following *H*. *pylori* eradication therapy.

## 2. Case Presentation

A 54-year-old male patient sought medical care for recurrent episodic headaches of 4-month duration occurring concomitantly with epigastric pain. He described his headaches as primarily affecting the bilateral temporal regions and “throbbing” in nature.

Laboratory tests including complete blood count, basic metabolic panel, liver function tests, and thyroid function tests were normal. The patient underwent a comprehensive headache workup including neurological examination, imaging studies (CT and MRI with contrast of the brain) and lumbar puncture. The results were normal and did not reveal any abnormality. Despite extensive diagnostic evaluations, the etiology of the headache remained unclear.

**Figure 1:**
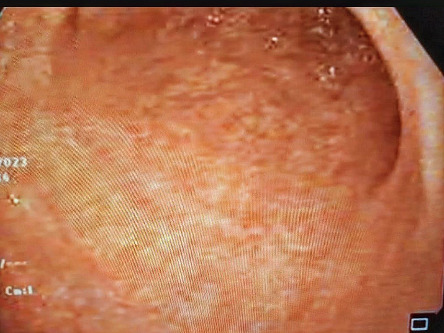
An esophagogastroduodenoscopy (EGD) under white light endoscopy revealing mosaic appearance of the gastric mucosa with diffuse hyperemia.

**Figure 2:**
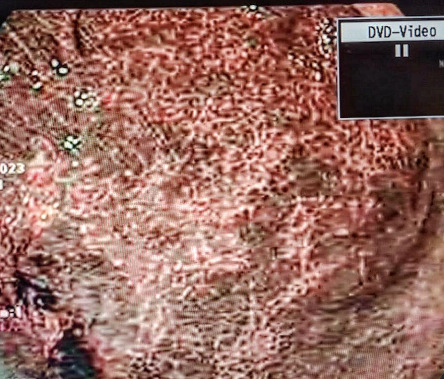
An EGD under narrow-band-imaging (NBI) demonstrating the absence of regular arrangement of collecting venules plus redness and mucosal swelling, indicating an infection with *H. pylori.*

Owing to persistent epigastric pain, an upper endoscopy was employed, revealing mosaic appearance of the gastric mucosa accompanied by diffuse hyperemia (Figure 1). Subsequently, an esophagogastroduodenoscopy (EGD) under narrow-band-imaging (NBI) was performed demonstrating the absence of regular arrangement of collecting venules plus redness and mucosal swelling, indicating an infection with *H. pylori* (Figure 2).

Multiple biopsies were taken from the gastric body, antrum, and incisura angularis. Pathology studies unveiled severe chronic gastritis, antrum predominant, along with reactive lymphoid follicular hyperplasia and diffuse lymphoid infiltrate with occasional lympho-epithelial lesions, corroborating a diagnosis of MALT lymphoma. H. *pylori* testing was positive. A diagnosis of *H*. *pylori*-positive MALToma was made.

**Figure 3:**
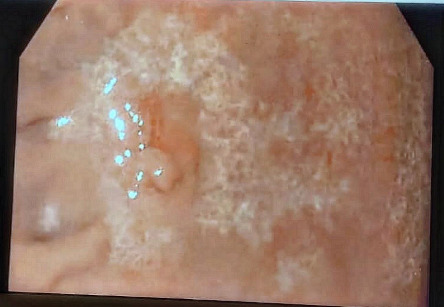
A follow-up EGD under white light endoscopy was performed 3 months after the completion of *H. pylori* eradication therapy, showing a white discoloration of the mucosa. This indicates atrophic changes of the mucosa following recovery from the lymphoma.

The patient was started on a 14-day bismuth-based quadruple therapy regimen for *H. pylori* eradication. A follow-up urea breath test was performed 3 months after the completion of *H. pylori* eradication therapy, confirmed the resolution of *H. pylori* infection. Repeated endoscopy shows a white discoloration of the mucosa (Figure 3). This finding indicates atrophic changes of the mucosa following the recovery from the lymphoma. Repeated biopsy results showed no evidence of residual lymphoma.

Following treatment for *H. pylori*, both the gastric MALToma and headaches resolved completely, suggesting a potential link between the two conditions. In other words, complete resolution of the patient's episodic headaches was attained, coinciding with the eradication of *H. pylori* and the resolution of the MALToma. Hence, this conjunction establishes a causality between headaches and *H. pylori* infection.

## 3. Discussion

MALT lymphoma is an extranodal marginal zone lymphoma. It is a slow-growing, low-grade form of non-Hodgkin lymphoma that develops from B cells. Patients with gastric MALToma may present with a wide range of symptoms ranging from epigastric discomfort to upper GI bleed causing iron-deficiency anemia. Rarely, it manifests with B symptoms of fever, night sweats, and weight loss [1]. Patients with these symptoms undergo an endoscopic procedure in about half of the cases, revealing an ulcerative lesion [2]. However, other macroscopic patterns may be visualized such as submucosal tumor, multiple erosion, cobblestone mucosa, partial fold thickening, and discoloration types [2].

Not all patients with MALT lymphoma have macroscopic changes of the gastric mucosa. It was reported that 9% of the cases of MALT lymphoma patients have normal or hyperemic gastric mucosa [3].

The definitive diagnosis of gastric MALT lymphoma is established by gastric biopsy [1]. Because of the patchy distribution of MALT lymphoma, it is important to have multiple biopsies from different sites of the stomach to exclude possible indolent lymphoma.

A plausible approach to enhance detection of *H. pylori* and premalignant mucosa using white light gastroscopy is to target mucosal biopsies to certain morphologies. A study categorized gastric mucosa into four morphologies and investigated each one's predictability for *H. pylori* status as follows: type 1 with a regular arrangement of collecting venules, type 2 with a cleft-like appearance, type 3 with a mosaic appearance, and type 4 with a mosaic appearance accompanied by focal or diffuse hyperemia (Table 1) [4]. Mucosa types 3 and 4 were more predictive of *H. pylori* infected mucosa [4]. Our patient exhibited gastric mucosa type 4 (Figure 1).

Another imagining technique that is used to enhance diagnosis of mucosa pathologies is narrow-band-imaging (NBI). Mucosal structures and vascular patterns can be visualized using NBI during gastroscopy [5]. In normal noninfected mucosa, a regular arrangement of collecting venules is seen on NBI [5]. In contrast, an absence of this pattern plus redness and mucosal swelling indicates an infection with *H. pylori* [5]. In our patient, the mucosa is depicted under NBI with noticeable swelling and redness.

The first-line treatment of MALT lymphoma is the eradication therapy for *H. pylori* for patients who are in early stages and tested positive for the pathogen [1]. Chemotherapy is used for patients with more advanced stage disease. Immunotherapy with rituximab is usually added to the chemotherapy regimen [1]. For patients with localized lymphoma who tested negative for *H. pylori,* radiation therapy is employed. Surgery is only indicated if the patient has complication related to the disease, such as pyloric stenosis, gastric wall perforation, or uncontrolled hemorrhage [1].

This case highlights the importance of considering GI pathology in patients presenting with unexplained headaches. The complete resolution of headaches following the treatment of gastric MALToma and *H. pylori* infection in this patient points to a potential link between the two conditions.

Several studies have found a high prevalence of *H*. *pylori* infection in patients suffering from migraines compared with the control group [6]. The migraine might be resulting from the activation of the immune system against the bacteria and the release of vasoactive substances and proinflammatory cytokines [6]. For example, patients with *H. pylori* infection exhibit elevated levels of IL-10, a cytokine that is similarly increased during migraine attacks [7].

In addition, gastrointestinal neuroendocrine cells synthesize and secrete 5-hydroxytryptamine, substance P, and vasoactive intestinal polypeptides in response to inflammation caused by *H. pylori* [6].

A study by Gasbarrini et al. described a correlation between patients having migraine with aura and an infection with specific strain of *H*. *pylori* carrying the cytotoxic associated gene A (CagA), type I [8]. These strains of bacteria are more potent in triggering the immune system, leading to an increased proinflammatory molecules release, causing systemic vasospasm [8]. The vasospasm in cerebral arteries leads to the aura that is associated with migraine [8].

Another explanation for the headache is the ability of the bacteria to release histamine. It has been proven that cultures of *H*. *pylori* produce histamine molecules [9]. This can be explained by the vasoactive role of histamine that can alter the blood brain barrier permeability and cause neurogenic inflammation.

The link between *H*. *pylori* and migraine is still not well established. However, the treatment of *H*. *pylori* for infected patients suffering from migraine has favorable outcome in decreasing the severity of the migraines as was exemplified in our case.

## 4. Conclusion

*H*. *pylori* can be a culprit behind the development of MALToma. In early stages, the cornerstone treatment for *H*. pylori-positive MALToma is antibiotics therapy in conjunction with PPIs, followed by a documentation of complete eradication of *H*. pylori. This article underscores the intimate relationship between the brain and the gut, whereby MALToma manifested as recurrent headaches. In our case, MALT lymhoma defied normality by emerging as recurrent headaches. Thus, physicians should include MALT lymphoma in their differential diagnosis when approaching a patient with headaches of unclear etiology. The link between *H*. *pylori* and migraine is still not well established. Thus, this article serves as an igniter to fuel further research to elucidate the causality between migraines and *H*. *pylori* infection.

## Figures and Tables

**Table 1:** Categorization of gastric mucosa into four morphologies.

Gastric mucosal morphology	Description
Type 1	Regular arrangement of collecting venules
Type 2	Cleft-like appearance
Type 3	Mosaic appearance
Type 4	Mosaic appearance + focal/diffuse hyperemia

## Data Availability

The data used to support the findings of this study are available from the corresponding author upon reasonable request.
